# The Spectrum of Postacute Sequelae of COVID-19 in Children: From MIS-C to Long COVID

**DOI:** 10.1146/annurev-virology-093022-011839

**Published:** 2024-08-30

**Authors:** Abigail S. Kane, Madeleine Godfrey, Magali Noval Rivas, Moshe Arditi, Alessio Fasano, Lael M. Yonker

**Affiliations:** 1 Children’s Hospital of Los Angeles, Los Angeles, California, USA; 2 Mucosal Immunology and Biology Research Center, Massachusetts General Hospital, Boston, Massachusetts, USA; 3 Department of Pediatrics, Division of Infectious Diseases and Immunology, Guerin Children’s, Cedars-Sinai Medical Center, Los Angeles, California, USA; 4 Infectious and Immunologic Diseases Research Center and Department of Biomedical Sciences, Cedars-Sinai Medical Center, Los Angeles, California, USA; 5 Department of Pediatrics, Massachusetts General Hospital, Boston, Massachusetts, USA; 6 Department of Pediatrics, Harvard Medical School, Boston, Massachusetts, USA

**Keywords:** COVID-19, MIS-C, PASC, long COVID, spike

## Abstract

The effects of SARS-CoV-2 infection on children continue to evolve following the onset of the COVID-19 pandemic. Although life-threatening multisystem inflammatory syndrome in children (MIS-C) has become rare, long-standing symptoms stemming from persistent immune activation beyond the resolution of acute SARS-CoV-2 infection contribute to major health sequelae and continue to pose an economic burden. Shared pathophysiologic mechanisms place MIS-C and long COVID within a vast spectrum of postinfectious conditions characterized by intestinal dysbiosis, increased gut permeability, and varying degrees of immune dysregulation. Insights obtained from MIS-C will help shape our understanding of the more indolent and prevalent postacute sequelae of COVID and ultimately guide efforts to improve diagnosis and management of postinfectious complications of SARS-CoV-2 infection in children.

## INTRODUCTION

Early in the COVID-19 pandemic, amid nationwide lockdowns and as we struggled to digest the repercussions of the emergence of SARS-CoV-2, a mysterious Kawasaki-like illness characterized by prolonged fever and severe multiorgan involvement began to be reported in children around the globe (1–4). Previously healthy children who were initially thought to be spared from COVID-19 were now requiring hospitalization and often even intensive care for a rare systemic hyperinflammatory response associated with recent SARS-CoV-2 exposure or mild infection. The first cases of this newly identified postviral syndrome were reported in the United Kingdom in late April 2020 (4). Children presented with unremitting fever, gastrointestinal symptoms, mucocutaneous involvement, and progression to cardiogenic shock in the setting of elevated inflammatory biomarkers, reminiscent of both Kawasaki disease and toxic shock syndrome. Additional cases quickly occurred in other countries, including the United States. New York, the first major city in the United States to experience the enormity of the pandemic, was also the first to report a case series of 95 children and young adults presenting to already overwhelmed emergency departments with complaints similar to those reported in Europe (5). While many patients presented 2–8 weeks after exposure to a family member with COVID-19, many were not aware that they had been infected, as they may have been asymptomatic or did not undergo testing due to limitations in polymerase chain reaction (PCR) availability. This illness was termed multisystem inflammatory syndrome in children (MIS-C), also known as pediatric inflammatory multisystem syndrome (PIMS). MIS-C/PIMS was ultimately recognized as an aberrant, postacute, hyperinflammatory response to SARS-CoV-2 that could occur after symptomatic COVID-19 or asymptomatic infection.

As the pandemic progressed and SARS-CoV-2 infection rates continued to rise, other forms of postacute sequelae of COVID-19 (PASC) emerged, with multiorgan involvement but a milder, more indolent, and prolonged inflammatory presentation. After the resolution of acute infection, patients frequently presented with new, lingering, or worsening symptoms such as severe fatigue, cognitive difficulties, persistently altered sense of taste and smell, dysautonomia, dyspnea, and exercise intolerance with no apparent cause. Although PASC was initially approached with skepticism, it has ultimately been recognized as a new entity encompassing hundreds of symptoms of varying severity resulting from persistent immune activation, occurring weeks to months after the resolution of acute SARS-CoV-2 infection. PASC is now recognized as a broad spectrum of disease, with MIS-C representing its most severe form. Indeed, early efforts aimed at defining the pathophysiologic mechanisms of MIS-C now inform an understanding of the pathology seen across the spectrum of PASC, including long COVID.

## ZONULIN-DEPENDENT LOSS OF GUT MUCOSAL BARRIER FUNCTION DRIVES MIS-C

**Figure 1:**
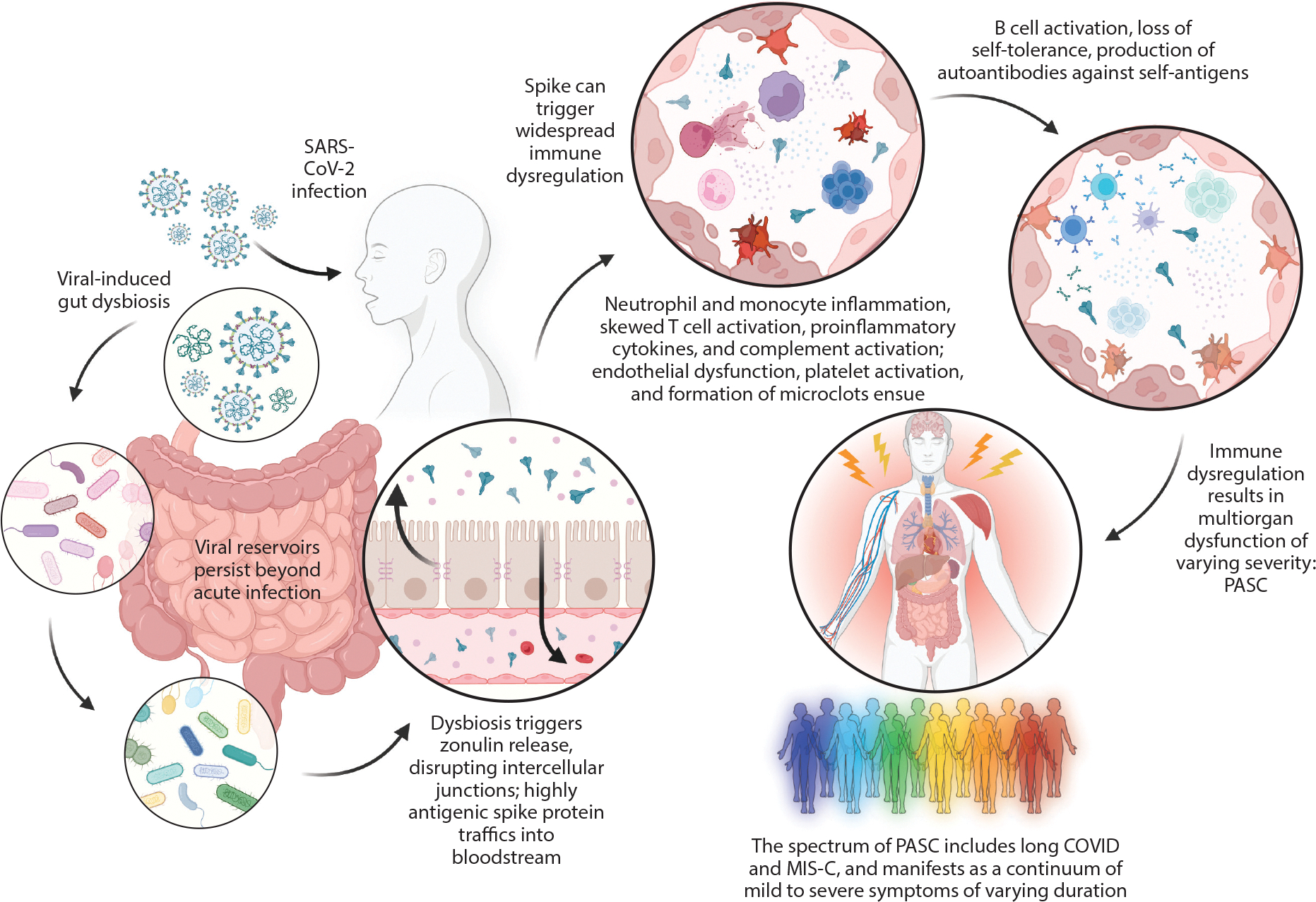
Mechanism driving PASC, including MIS-C and long COVID. Abbreviations: MIS-C, multisystem inflammatory syndrome in children; PASC, postacute sequelae of COVID-19. Figure adapted from images created with BioRender.com.

MIS-C occurs as a consequence of an exaggerated and dysregulated activation of the immune system in response to circulating SARS-CoV-2 spike antigen. Although knowledge about specific immune activation pathways continues to evolve, MIS-C is instigated by gut dysbiosis and impaired intestinal mucosal barrier function. Children with MIS-C have prolonged SARS-CoV-2 RNA shedding in the stool despite the resolution of acute COVID-19 symptoms, suggesting continued viral infection in the gastrointestinal tract (6). As with other viruses, SARS-CoV-2 reservoirs in the gut can impair cellular function and alter the composition of the gut microbiota, resulting in intestinal dysbiosis (7–9). This dysbiosis, in turn, establishes an environment that favors the disruption of the mucosal barrier, notably by triggering the release of zonulin, a molecule involved in the physiologic regulation of intestinal tight junctions, and thus allowing for paracellular trafficking of the highly antigenic SARS-CoV-2 spike protein into the bloodstream (6, 10, 11). Support for this mechanism comes from the observation that children with MIS-C have spike antigenemia and serum markers reflecting increased intestinal permeability despite the absence of nasopharyngeal detection of SARS-CoV-2 using PCR (6). Similar pathologic mechanisms appear to drive other forms of PASC, with gastric reservoirs of SARS-CoV-2, increased markers of gastrointestinal permeability, and spike antigenemia detected in persons with long COVID (12–14) (Figure 1).

**Figure 2:**
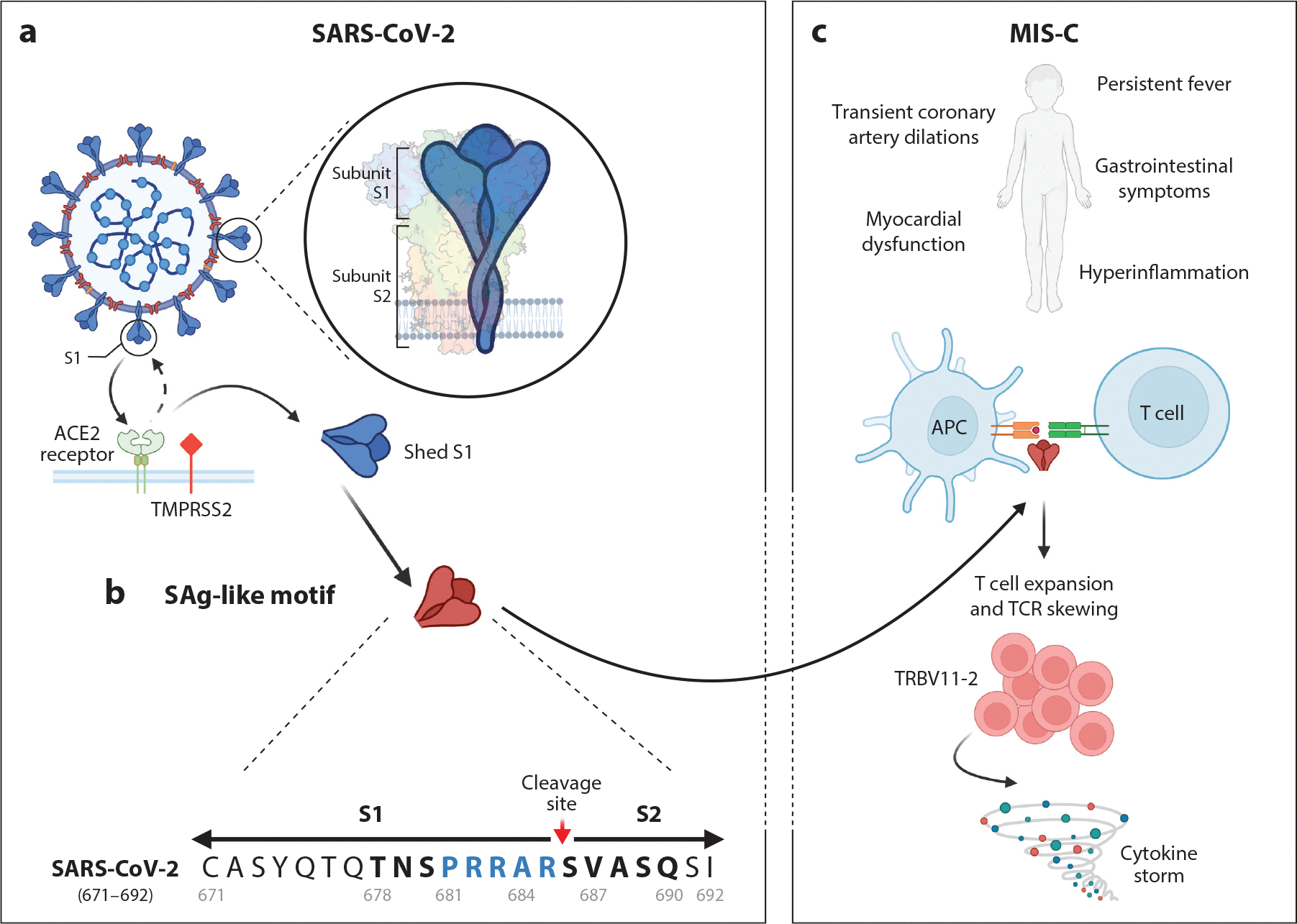
A SAg-like motif in SARS-CoV-2 spike protein. (*a*) SARS-CoV-2 spike (*blue*) proteins expressed at the surface of SARS-CoV-2 are composed of two subunits, S1 and S2. Spike proteins interact with the host cell ACE2 receptor (*green*) and transmembrane protease TMPRSS2 (*red*). After binding to ACE2, spike proteins are cleaved by proteases (furin and TMPRSS2) at the S1/S2 junction, leading to S2 membrane fusion and the shedding of S1. (*b*) SARS-CoV-2 S1 has a unique insertion of four amino acids, PRRA, adjacent to the S1/S2 cleavage site (R_685_-S_686_). This polybasic PRRA insert is part of a motif (*bolded*) whose sequence and structure highly resemble a segment of a bacterial SAg, Staphylococcal enterotoxin B (17). (*c*) Studies have reported a unique TCR repertoire in MIS-C patients, consistent with a TCR skewing typical of the reaction to SAgs, which for MIS-C patients is characterized by the expansion of TRBV11–2 cells (18, 20, 21, 23). This TRBV11–2 cell T cell expansion also correlates with disease severity and hyperinflammation in MIS-C patients (18, 21). Abbreviations: ACE2, angiotensin-converting enzyme 2; APC, antigen-presenting cell; MIS-C, multisystem inflammatory syndrome in children; PRRA, P_681_RRA_684_; SAg, superantigen; TCR, T cell receptor; TMPRSS2, transmembrane serine protease 2; TRBV11–2, T lymphocytes containing Vβ gene 11–2. Figure adapted from An In-depth Look into the Structure of the SARS-CoV2 Spike Glycoprotein by BioRender.com (2024), retrieved from https://app.biorender.com/biorender-templates.

Many downstream effects occur once the spike protein encounters subepithelial immune cells. SARS-CoV-2 spike harbors a superantigen (SAg)-like motif adjacent to the S1/S2 cleavage site that is capable of nonspecifically binding to the variable domain on β chains of T cell receptors (TCRs) (Figure 2). SAg binding to TCRs leads to overactivation of T cells and cytokine storm, which are both observed in severe COVID-19 and MIS-C (15–17). The SARS-CoV-2 SAg-like motif could also contribute to the dysregulated immune response and autoimmune manifestations associated with long COVID (15, 16). In general, SAgs skew the TCR repertoire due to clonal expansion of T lymphocytes containing certain variable β (Vβ) chains capable of ample antigen binding. Children with MIS-C have a transient clonal expansion of T lymphocytes containing Vβ gene 11–2 (TRBV11–2) (Vβ 21.3), which is capable of interacting with the spike SAg-like motif, and the degree of clonotype expansion correlates with proinflammatory cytokine production and MIS-C severity (17–20). Additionally, TRBV11–2 skewing is associated with specific human leukocyte antigen I (HLA-I) alleles (A02, B35, and C04), which may reflect a genetic predisposition to the development of MIS-C (21, 22). Collectively, the high-affinity binding motif for TCR adjacent to the spike S1/S2 cleavage site in conjunction with the expansion of TRBV11–2 T cells supports the idea that spike, particularly the SAg-like motif, is responsible for T cell activation and subsequent inflammatory response in MIS-C.

## IMMUNE DYSREGULATION DRIVES HYPERINFLAMMATION IN MIS-C

Although MIS-C is associated with lymphopenia and reduction of both CD4 and CD8 T cell subsets, the remaining T lymphocytes are highly active, with increased expression of CD38 and major histocompatibility complex class II receptor HLA-DR (20, 23, 24). T cell activation in MIS-C induces a type II interferon (IFN) response (IFN-γ) that further activates T cells, dendritic cells, and macrophages that collectively drive the culmination of a cytokine storm consisting of IFN-γ, tumor necrosis factor-α (TNF-α), interleukin (IL)-1β, IL-6, IL-8, IL-10, IL-17, and IL-18 (24–26). Even within MIS-C, a range of symptom severity is observed, with more severe forms [i.e., requiring intensive care unit (ICU) admission] displaying more profound cytokine release (27).

B cell–mediated responses are also aberrant in MIS-C. Despite peripheral lymphopenia with decreased total, effector, and memory B cells, children with MIS-C have increased B cell activation (including autoreactive B cell activation) and immunoglobulin (Ig) production with increased CD86 expression and decreased expression of HLA-DR and CD25, reflecting distorted antigen-presenting capacity (23). Furthermore, multiple stimuli, including neutrophilic expression of B cell–activating factor and cytokine stimulation, drive loss of self-tolerance and autoreactive B cell expansion (18). Patients with MIS-C can harbor myriad IgA and IgG autoantibodies against self-antigens in the endothelium and various organs, including anti-IL-1RA, which leads to excessive IL-1β signaling and contributes to widespread inflammation (28, 29). Many of these autoantibodies have been associated with chronic autoimmune disorders such as type 1 diabetes mellitus, Sjögren syndrome, and systemic lupus erythematosus (30, 31).

MIS-C is associated with a profound activation of innate immune cells. Expansion of classical monocyte subsets and decreases in nonclassical and intermediate subsets drive cytokine production (e.g., IL-6 and TNF-α) and likely contribute to endothelial cell injury and dysfunction (32–34). Spike-immune complexes activate neutrophils to further exacerbate inflammation and cytokine production, driving intravascular neutrophil extracellular trap formation, release of cytotoxic granules, and production of reactive oxygen species (35). Activated monocytes and neutrophils overexpress surface marker CD64, which contributes to increased interaction with autoantibodies and high-affinity human IgG receptor Fc gamma receptor I, resulting in further inflammation and end-organ damage.

MIS-C is also characterized by complement activation with enhanced expression of both classical (C1qA, C1qB, and C1qC) and alternative complement pathway components (18). Autoantibody immune complexes, in part, trigger the complement activation in MIS-C, and differences in plasma complement levels between mild and severe MIS-C suggest differences in consumption due to more robust activation of complement pathways in children with more severe phenotypes requiring ICU admission (18).

## SHARED PATHOPHYSIOLOGIC MECHANISMS SUGGEST LONG COVID IS A MORE INDOLENT VERSION OF MIS-C

The extensive efforts to define hyperinflammatory profiles of MIS-C have informed the pathophysiologic mechanisms of long COVID. Indeed, like MIS-C, long COVID is characterized by gastrointestinal reservoirs of SARS-CoV-2, which can last over one year after the acute infection (12, 36, 37). Increased zonulin and spike antigenemia are also detected in long COVID, as are signs of immune complex activation of neutrophils, with evidence of ongoing, hyperactive, or altered SARS-CoV-2-specific T cell– and B cell–specific responses, including the possible development of autoreactive T cells and antibodies with cross-reactive autoimmunity (12, 14, 38–40). This shared pattern of persistent viral reservoirs, intestinal permeability, spike antigenemia, and dysregulated inflammation highlights that MIS-C and long COVID represent a continuum of the PASC disease spectrum with a shared mechanistic pathway (Figure 1).

**Figure 3:**
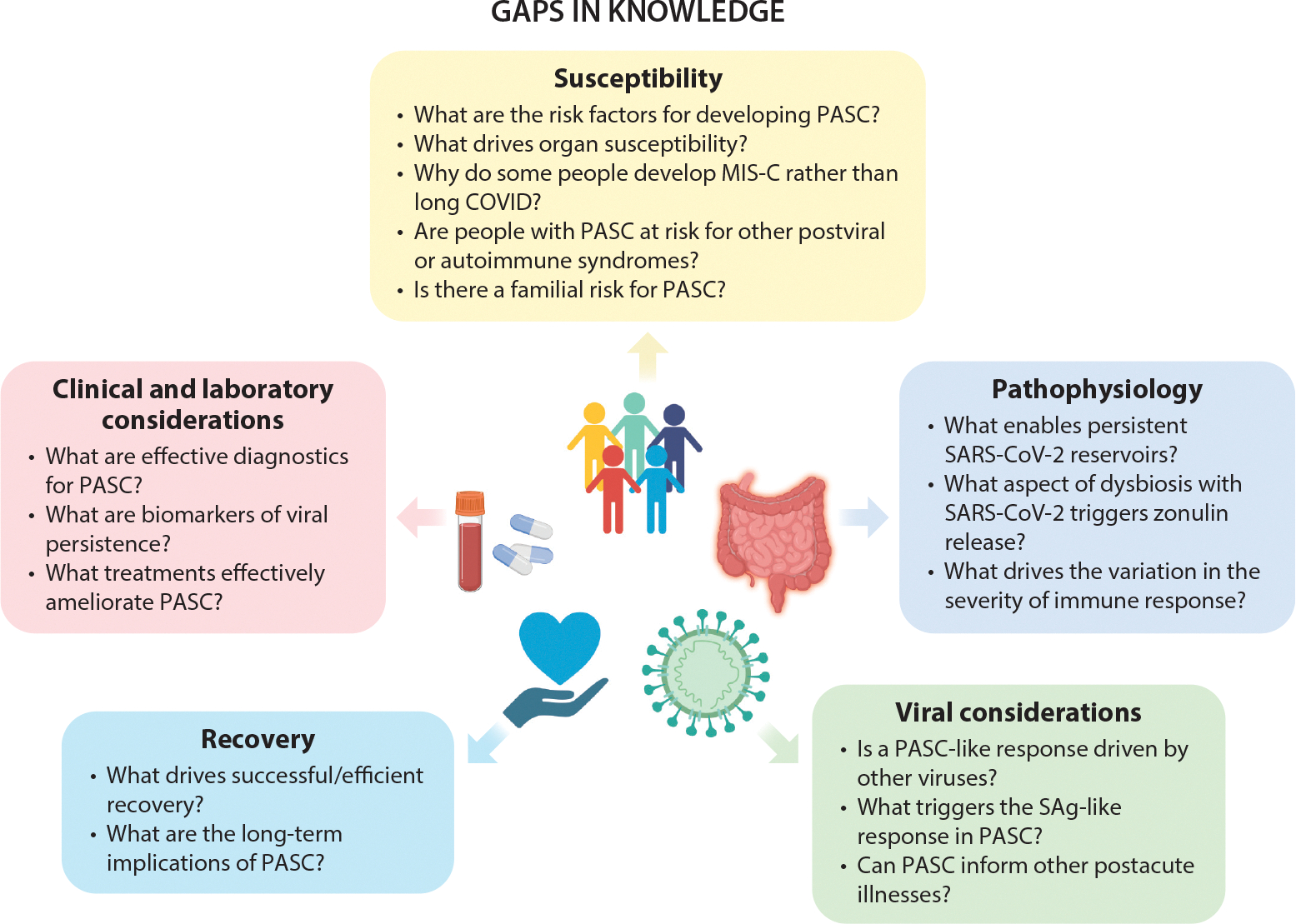
Gaps in knowledge regarding MIS-C and long COVID. Abbreviations: MIS-C, multisystem inflammatory syndrome in children; PASC, postacute sequelae of COVID-19; SAg, superantigen. Figure adapted from images created with BioRender.com.

Other inflammatory diseases associated with impaired intestinal barrier function and increased gut permeability do not necessarily predispose children to long COVID or MIS-C, suggesting that a combination of factors drives susceptibility to these illnesses. Obesity is associated with a twofold increase in risk of MIS-C and is linked with severe disease course and a greater risk of incomplete recovery from MIS-C (41, 42). The proinflammatory state that characterizes obesity has been proposed to contribute to widespread endothelial, adipocyte, and macrophage activation, which in turn can result in more severe disease following acute illness (41–43). However, this process needs to be further understood. Similarly, much remains to be understood about the role of viral features and host susceptibility as determinants of the postacute disease spectrum, including what factors distinguish the development of severe MIS-C as compared to long COVID (Figure 3). As occurs with other immune-mediated diseases, MIS-C develops when genetic and immunologic predispositions, including HLA type, intersect with external factors (44, 45), and a greater understanding of the clinically identifiable risk factors for progression to MIS-C and long COVID is of utmost importance to guide prevention.

## PROTEAN SYMPTOMS AND LACK OF DIAGNOSTIC TESTS CHALLENGE THE DETECTION OF MIS-C AND LONG COVID

To date, more than 700 million confirmed cases of COVID-19 have been reported worldwide, with more than 100 million cases in the United States (46). It is now estimated that more than 96% of children have been infected by SARS-CoV-2 based on serologic testing (47). MIS-C is a rare complication that occurs in 1 in 3,000 to 4,000 children and young adults with SARS-CoV-2 exposure, carrying a 1–3% mortality rate (48, 49). As of November 2023, a total of 9,558 cases of MIS-C have been reported in the United States (48), although this number is likely an underestimate given its protean, nonspecific manifestations, which can be seen in several other infectious and inflammatory diseases, compounded by the lack of objective diagnostic tests.

Because MIS-C occurs an average of 4 weeks following SARS-CoV-2 exposure, epidemiologic surveillance has consistently shown peaks in new cases of MIS-C following waves of SARS-CoV-2 infection (48). However, the incidence of MIS-C has varied throughout the pandemic (Table 1). MIS-C was most prevalent in 2020 when the ancestral and Delta variants (B.1.617.2) were predominant, infection rates were higher, and vaccines were not yet available for children. Additionally, the clinical severity of MIS-C and incidence of complications have decreased with subsequent SARS-CoV-2 variants, likely due to a combination of factors, including increased natural and vaccine-induced immunity (50). Furthermore, some children who met all other diagnostic criteria for MIS-C experienced a milder form of the disease and did not require hospitalization, suggesting the existence of a spectrum of disease severity to these postviral syndromes associated with SARS-CoV-2 (51). Ultimately, the epidemiologic trends of MIS-C and pediatric long COVID remain challenged by a lack of clarity in diagnostic testing. Without high clinical suspicion and rigorous patient tracking combined with organized research efforts focused on studying pediatric PASC, including MIS-C, clinical advances will be unattainable.

## NEW STRATEGIES ARE REQUIRED TO IDENTIFY AND EFFECTIVELY TREAT PASC

All conditions within the PASC spectrum are diagnoses of exclusion requiring a high degree of clinical suspicion. While evidence of prior exposure to SARS-CoV-2 (e.g., nucleocapsid antibodies or report of prior positive PCR or antigen test) is required for MIS-C diagnosis, the role of SARS-CoV-2 antibodies is no longer clear and may change moving forward, as estimates suggest that the virus has infected more than 96% of children worldwide. Furthermore, inflammatory markers of MIS-C, including C-reactive protein, ferritin, and monocyte distribution width, are nonspecific (52–54), and new biomarkers are, therefore, urgently needed to detect MIS-C and other forms of pediatric PASC.

Some individuals with long COVID may exhibit higher levels of antibodies against SARS-CoV-2, evidence of reactivated humoral responses against other viruses such as Epstein Barr virus, distinct circulating myeloid and lymphocyte populations, lower cortisol levels (55), and, potentially, decreased serotonin levels (56). Like with MIS-C however, these tests are nonspecific.

Cytokine profiles, TCR profiling, gene expression profiles, cell-free DNA circulating in peripheral blood, and ultrasensitive single molecule spike antigen blood assays have emerged as promising diagnostic tools, but they are not yet available in clinical settings (14, 57, 58). As our understanding of pathophysiology expands, new biomarkers also may be developed to identify disease on the PASC spectrum more effectively, and the clinical role of assays to quantify zonulin, spike, T cell subsets, presence of microclots, or identification of specific HLA haplotypes is yet to be determined (Table 2).

The management of post-COVID disorders requires a multidisciplinary team, and therapeutic needs vary based on disease severity. The treatment for MIS-C mirrors therapeutic strategies for Kawasaki disease aimed at reducing inflammation using immunomodulators, such as intravenous immunoglobulin, glucocorticoids, or both (59). Options for the management of refractory MIS-C include high-dose anakinra, a recombinant human IL-1 receptor antagonist, and infliximab, a monoclonal antibody that binds TNFα, thus preventing its proinflammatory effects (60). In contrast to MIS-C, the management of other disorders along the PASC spectrum is not as well established. Given the vast array of reported postacute ailments, treatment is largely aimed at symptom reduction. Immunomodulators and antivirals have been used to target immune dysregulation, but placebo-controlled trials have yet to determine whether these approaches are effective in the treatment of disorders along the PASC spectrum beyond MIS-C (61).

Given the prothrombotic state associated with MIS-C, thromboprophylaxis with low-dose aspirin is recommended for all patients with MIS-C unless they present with risk factors for bleeding, including thrombocytopenia or reduced fibrinogen levels (54, 62). Platelet activation with microthrombus formation is also associated with PASC in adults, and dual antiplatelet therapy (e.g., aspirin in combination with clopidogrel) together with direct oral anticoagulation (e.g., apixaban) has been tested, resulting in anecdotal improvement of symptoms (63, 64). Larger, placebo-controlled trials are needed, and it remains to be determined whether treatment strategies used for MIS-C could be modified to benefit people with long COVID.

New investigational treatments for MIS-C include remestemcel-L, a mesenchymal stromal cell therapy agent, and larazotide, a zonulin receptor antagonist. Remestemcel-L stimulates anti-inflammatory cytokines and growth factors that promote tissue repair and restore endothelial function, and case reports suggest improvement in inflammatory profiles and left-ventricular ejection fraction (a measure of cardiac function) in children who did not respond appropriately to traditional treatment (65). Larazotide is an investigational drug that targets the suspected root cause of MIS-C, enteral antigen absorption, rather than the subsequent inflammatory response. Larazotide binds to zonulin receptors on the apical surface of enterocytes, restoring the mucosal gut barrier and preventing further paracellular passage of SARS-CoV-2 spike antigen into the bloodstream (6, 66). Targeted treatments for the PASC spectrum are urgently needed and require larger, placebo-controlled clinical trials to assess efficacy in both children and adults.

## LONG-TERM EFFECTS OF PEDIATRIC PASC, INCLUDING MIS-C, ARE YET TO BE DETERMINED

Appropriate management of MIS-C is generally associated with acute symptom resolution and short-term restoration of gross organ function (67–72). For example, although patients who present with cardiac involvement may develop severe left ventricular dysfunction and cardiogenic shock requiring extracorporeal membrane oxygenation (ECMO), short-term follow-up after completion of immunomodulator treatment is not associated with persistently reduced left ventricular ejection fraction or myocardial fibrosis (73, 74). However, it is possible that the widespread endothelial dysfunction and underlying disordered immune response associated with MIS-C could contribute to an increased risk of chronic inflammatory and autoimmune conditions later in life (75). Up to 25% of children with MIS-C can experience prolonged symptoms similar to those of long COVID, including reduced exercise tolerance and new-onset neuropsychiatric symptoms (e.g., irritability, anxiety, and brain fog) after resolution of the acute phase of their disease (67, 68, 74, 76). These observations further suggest that MIS-C and the many symptoms of long COVID are not entirely distinct but rather part of a continuum.

Although the risk of developing MIS-C is low, children, like adults, can also develop more indolent forms of PASC, including long COVID. While mortality rates across the PASC spectrum are fortunately much lower than those of MIS-C, the morbidity and economic burden of PASC warrant attention. It is estimated that PASC has affected more than 65 million people worldwide, including up to 25% of children infected with SARS-CoV-2, and currently costs the US economy $3.7 trillion annually with medical expenses accounting for $528 billion (39, 77, 78). In addition to the economic burden, the long-term repercussions of pediatric PASC in general, compounded by disruptions in education, social engagement, and neurodevelopment, will not be fully realized for years to come.

## VACCINES AS TOOLS FOR PREVENTION OF MIS-C AND OTHER PASC

Because SARS-CoV-2 exposure is the leading risk factor for developing MIS-C and PASC, vaccines can prevent these conditions. Of the prophylactic strategies known, vaccines are the most effective method for reducing viral transmission and preventing infection, thus decreasing the risk of developing MIS-C (79, 80). Importantly, maternal immunization during pregnancy results in strong passive immunity that can also help prevent MIS-C in infants under 6 months of age who are not yet eligible for COVID-19 vaccines (81, 82). This overall protective effect is evidenced by a decline in MIS-C incidence since COVID-19 vaccines became available (79, 80).

Despite their overall protective efficacy, vaccines do not provide immunity in all cases. Both breakthrough infection and subsequent MIS-C can occur due to waning immunity and sequence changes in SARS-CoV-2 variants. However, vaccination can decrease the severity of MIS-C, as previously immunized children with MIS-C experience a milder disease course than unvaccinated children that is characterized by less severe inflammation, shorter time to recovery, and decreased risk for requiring mechanical ventilation and ECMO support (79).

There have been reports of MIS-C following the administration of SARS-CoV-2 messenger RNA (mRNA) vaccines. This phenomenon, termed MIS-V (multisystem inflammatory syndrome following vaccination), is thought to occur in less than 2 per 1,000,000 recently immunized children, which is significantly less common than the already infrequent occurrence of MIS-C (79). Importantly, the temporal association between MIS and vaccination does not imply causation, particularly given that inadvertent, unrecognized SARS-CoV-2 exposure around the time of vaccination is always a possibility, especially in children in whom infection may be asymptomatic. In a world where SARS-CoV-2 is now ubiquitous, it is becoming increasingly difficult to differentiate between prior infection and recent inadvertent exposure with an asymptomatic course based on the presence of nucleocapsid antibodies by serologic testing. Nonetheless, similar to how the benefits of COVID-19 vaccination outweigh the low risk of mRNA vaccine-associated myocarditis (83), the possibility of developing a systemic hyperinflammatory response to vaccination is extremely rare, and the advantages of immunization are many, including protection against severe COVID-19 and MIS-C.

## CONCLUSION

Since the start of the pandemic, MIS-C cases have dropped dramatically, which may be related to vaccination, evolution of the virus, a gradual shift from the more severe and acute MIS-C to a more indolent and long-standing long COVID syndrome, ambiguity of diagnosis and reporting, or a combination of these variables. It is essential that we continue the effort to build our understanding of both MIS-C and long COVID symptoms along the PASC spectrum to facilitate diagnosis, guide treatment, and identify and prevent the long-term health repercussions of these inflammatory conditions in children.

## Figures and Tables

**Table 1:** Reported COVID-19 cases, confirmed MIS-C cases, and confirmed long COVID cases over time (84–88)

Dates	Variant	Reported COVID-19 cases	COVID-19 patients developing MIS-C (%)	COVID-19 patients developing long COVID (%)
10/5/20–8/1/22	Alpha	1,208,988	0.066–0.13	1.0
10/5/20–10/30/23	Delta	4,612,262	0.033–0.050	0.5–10.8
8/1/21–10/30/23	Omicron	7,156,115	0.008–0.0084	0.2–4.5

Abbreviation: MIS-C, multisystem inflammatory syndrome in children.

**Table 2:** Biomarkers for diagnosis of MIS-C and long COVID

Condition	Commonly used diagnostic tools	Novel diagnostic tools
MIS-C	Complete blood count (lymphopenia, thrombocytopenia, leukocytosis with neutrophilia, elevated neutrophil lymphocyte count, monocyte anisocytosis) Complete metabolic panel (hyponatremia, hypoalbuminemia, elevated aspartate aminotransferase and alanine aminotransaminase) CRP, erythrocyte sedimentation rate, ferritin, fibrinogen, lactate dehydrogenase, D-dimer Troponin, brain natriuretic protein, N-terminal prohormone of brain natriuretic protein Urinalysis and urine microscopy (hematuria, low-grade proteinuria, and/or sterile pyuria) Transthoracic echocardiogram SARS-CoV-2 polymerase chain reaction and nucleocapsid antibodies	Cytokine profiles Gene expression profiles, cell-free circulating DNA, human leukocyte antigen haplotypes Serum zonulin Serum spike ultrasensitive single molecule assay Microclots T cell receptor flow cytometry Serum cortisol Epstein-Barr virus serology
Long COVID	Symptom-based and highly variable Diagnosis of exclusion	

Although case definitions have been refined over time, the US Centers for Disease Control and Prevention defines MIS-C as an illness characterized by the presence of subjective or documented fever ≥38.0°C, systemic inflammation (CRP ≥ 3.0 mg/dl), new-onset involvement of at least two organ systems, and disease severity requiring hospitalization in the absence of a more likely diagnosis. The listed diagnostic tools aid in identifying organ involvement. Although there are no specific diagnostic criteria for long COVID, it is defined as new, returning, or ongoing health problems that persist 4 weeks after infection. New biomarkers could help integrate the diagnosis of diseases along the postacute sequelae of COVID-19 spectrum. Abbreviations: CRP, C-reactive protein; MIS-C, multisystem inflammatory syndrome in children.
